# Screening for sleep apnoea risk in testosterone-treated transgender men

**DOI:** 10.3389/fneur.2023.1289429

**Published:** 2023-12-04

**Authors:** Bára Šnobrová, Kristina Burdová, Vladimír Weiss, Karel Šonka, Petr Weiss

**Affiliations:** ^1^Department of Neurology and Centre of Clinical Neuroscience, First Faculty of Medicine, Charles University and General University Hospital, Prague, Czechia; ^2^Department of Endocrinology, Poliklinika Modrany, Prague, Czechia; ^3^Institute of Sexology, General University Hospital, Prague, Czechia; ^4^Department of Psychology, Faculty of Arts, Charles University, Prague, Czechia

**Keywords:** snoring, testosterone, transgender men, Berlin Questionnaire, sleep apnoea

## Abstract

**Introduction:**

Obstructive sleep apnoea (OSA) is more prevalent in men. Several studies suggested that higher testosterone levels were associated with a greater risk of OSA. We aimed to determine whether testosterone administration in transgender men would accentuate symptoms of OSA.

**Methods:**

The study involved 94 adult people undergoing a female-to-male transition with testosterone administration. The participants answered the Berlin Questionnaire (BQ) and a separate question on snoring before starting testosterone treatment and after at least 1 year of being on testosterone treatment.

**Results:**

A higher proportion of participants at the follow-up answered positively to the first category of BQ devoted to snoring. A lower proportion of participants at follow-up answered positively to the second category of BQ devoted to tiredness. The percentage of subjects with a high risk of sleep apnoea, according to BQ, and of those who answered the question on snoring positively did not change significantly.

**Conclusion:**

An increased number of transgender men who reported snoring in BQ after testosterone administration indicate a higher risk of OSA development.

## Introduction

Obstructive sleep apnoea (OSA) is a condition caused by repeated episodes of upper airway collapse and obstruction during sleep. The prevalence of symptomatic OSA is ~2%−4% in men and 1%−2% in women. The etiology of OSA is multifactorial, and risk factors include obesity, male sex, age, menopause, fluid retention, adenotonsillar hypertrophy, and smoking (1).

Testosterone is the primary sex hormone and anabolic steroid in men. Therefore, testosterone therapy can be used as a treatment for many conditions (2) and is also an effective treatment for transgender people to induce the desired physical changes of the gender they identify with (3). Prescribing gender-affirming hormonal therapy to transgender men not only induces desirable physical effects but also benefits their mental health (4). In transgender men, testosterone therapy is aimed at achieving cisgender male serum testosterone to induce virilisation (4).

Several, but not all, studies have indicated that higher testosterone levels were associated with lower sleep intensity and a greater risk of OSA in men (5, 6). OSA due to testosterone treatment was reported in two transgender men (7). A recent report of six transgender men suffering from OSA shows that sleep-disordered breathing decreases the quality of life of individuals after the female-to-male transition, but there is no evidence of a relationship between the transition and sleep-disordered breathing (8).

Because of the well-documented associations between OSA and male sex, we aimed to determine whether testosterone administration would accentuate subjective symptoms of OSA.

## Methods

The study involved 94 adult people undergoing female-to-male transition, receiving intramuscular testosterone with a target testosterone plasma level of 10–30 nmol/L.

The participants completed Berlin Questionnaire (BQ) and provided a response to a question on respiratory arrests in sleep before starting their testosterone treatment. They were asked to respond to the same items during the testosterone treatment after 12 months or later, and we received 81 fully completed BQ and 86 answers to the question on respiratory arrests in sleep.

The BQ is a self-administered questionnaire that was developed to identify subjects with OSA in primary care settings (9). For the BQ, the overall result of the questionnaire was evaluated, and then, its three individual categories were evaluated separately. The BQ includes 10 items, of which five are related to snoring (category 1, items 1–5); three concern tiredness and sleepiness, with a sub-question about sleepiness while driving a motor vehicle (category 2, items 6–8); and two items related to the patient's history of hypertension and/or BMI >30 kg/m^2^ (category 3, items 9 and 10) (10). The result of each category was evaluated as either positive or negative. Two or more positive categories were considered as a risk of OSA.

Quantitative data (BMI, age) were compared using the paired *t*-test. Binary data (respiratory arrest and total BQ result and individual categories of BQ) were compared using the chi-squared test. Only participants who responded during the follow-up were included in the statistics.

The study was approved by the ethical committee of the General University Hospital, Prague, Czech Republic, and all participants agreed with the participation in the study by signing the informed consent form.

## Results

The results concerning BQ are displayed in Table 1. The proportion of participants with a high risk of sleep apnoea according to BQ has not changed during testosterone treatment. The proportion of participants labeled as positive in the first BQ category devoted to snoring increased and the proportion of subjects labeled positive in the second BQ category devoted to tiredness and sleepiness declined. The proportion of subjects labeled positive in the third BQ category has not changed. BMI and age did not change significantly during the observation of BQ results either.

**Table 1:** Results of the Berlin Questionnaire (number of respondents: 81).

	**Entry**	**Follow-up**	***p*-value**
Age—years (SD)	23.7 (±6.9)	24.9 (±6.9)	<0.001
BMI (SD)	25.7 (±7.2)	26.3 (±5.5)	n.s.
BQ sleep apnoea high risk (N, %)	11 (8.9%)	8 (6.5%)	n.s.
BQ first category (N, %)	9 (7.3%)	19 (15.4%)	0.038
BQ second category (N, %)	21 (17.1%)	9 (7.3%)	0.016
BQ third category (N, %)	19 (15.3%)	18 (14.6%)	n.s.

BQ, Berlin questionnaire; SD, standard deviation; n.s., non-significant; N, number.

The number of participants who answered positively to the question regarding respiratory arrests at entry was 4 (3.4%) and 7 (6.0%) at the follow-up (non-significant—n.s.). The mean age of respondents of this question was 23.5 (±6.8) years at entry and 24.7 (±6.8) years at the follow-up (*p* < 0.001). Their BMI at entry was 25.6 (±7.0) and 26.3 (±5.3) respectively (n.s.).

## Discussion

To our best knowledge, this is the first study to discuss sleep apnoea symptoms within the first months of testosterone treatment during the transgender female-to-male transition. Responses of the BQ first category devoted to snoring were more frequently positive when the subjects were under testosterone treatment. On the contrary, responses of the second BQ category devoted to fatigue were less frequently positive when the subjects were under treatment. The proportion of positive cases of the third BQ category devoted to hypertension remained unchanged as well as the proportion of participants at high risk of sleep apnoea according to total result of BQ. The proportion of subjects answering that they had sleep respiratory arrests was not significantly different despite the percentage doubled under the treatment.

Snoring is a highly prevalent condition associated with OSA and clinically indicates the risk of OSA (11). The increased number of subjects reporting snoring after 14 months of testosterone treatment may support the hypothesis that transgender men are at a higher risk of developing OSA.

In this case, the pathophysiology of worsening of breathing during sleep is not clear, it is not associated with any increase in BMI. In terms of disturbance in breathing during sleep, we suppose that the increase of subjects reporting snoring within such a short time span is not a result of an increase in age.

The collapse of the upper airway is the hallmark of apnoea and hypopnoea, and it occurs mostly due to the abnormalities in the upper airway. The role of testosterone in this phenomenon is not well-known. It is reported that abnormalities present in the orofacial skeleton may induce OSA (12), during the testosterone treatment no changes occur in the orofacial skeleton. Thus, aggravated snoring during testosterone treatment may be related to soft tissue changes or to the impairment of the pharyngeal and parapharyngeal muscle tone during sleep. These potential upper airway changes are related to testosterone itself or to lower levels of oestrogens, which are considered a protective factor of undisturbed breathing in sleep (13).

The lower occurrence of fatigue in transgender men can be explained by several factors. Prescribing gender-affirming hormonal therapy in transgender men not only induces desirable physical effects but also benefits mental health (4). Moreover, higher energy levels reflect better emotional health (14). Also, there may be a direct association between testosterone and the level of energy, which is commonly reported in men (15).

The study has several limitations. First, since the results of this study are based on a single questionnaire and one separate question, we did not track other sleep parameters. However, a recent study with a large sample size showed that testosterone treatment in transgender men does not substantially change the subjective assessment of sleep quality (16). Second, we did not collect the information on how many participants had bedpartners or other people that were able to witness the sleep disturbances of the participant. Third, the pattern of snoring has not been not discussed in detail since we could not assess the risk ratio according to snoring intensity, as in the study by Sowho et al. (11). Finally, the information on the general health and medication status of the participants is not included.

Based on our results, the growing number of transgender men seeking professional care (17, 18), and the prevalence of higher sleep disorders suspected in the transgender population (19), we recommend more elaborate longitudinal studies to determine whether there is a relationship between testosterone and the development of OSA.

## Conclusion

The increased number of transgender men reporting snoring after exogenous testosterone administration supports the hypothesis that testosterone administration may accentuate the risk of OSA.

## Data availability statement

The raw data supporting the conclusions of this article will be made available by the authors, without undue reservation.

## Ethics statement

The studies involving humans were approved by Ethics Committee of General University Hospital in Prague. The studies were conducted in accordance with the local legislation and institutional requirements. The participants provided their written informed consent to participate in this study.

## Author contributions

BŠ: Conceptualization, Data curation, Formal analysis, Investigation, Methodology, Writing—original draft, Writing—review & editing. KB: Formal analysis, Writing—review & editing. VW: Conceptualization, Investigation, Writing—review & editing. KŠ: Conceptualization, Supervision, Writing—original draft, Writing—review & editing, Funding acquisition, Investigation, Methodology. PW: Conceptualization, Investigation, Writing—review & editing.
